# Experiences managing behavioral symptoms among Latino caregivers of Latino older adults with dementia and memory problems: a qualitative study

**DOI:** 10.1186/s12877-024-05323-4

**Published:** 2024-08-31

**Authors:** Michelle S. Keller, Nathalie Guevara, Jose-Armando Guerrero, Allison M. Mays, Sara G. McCleskey, Carmen E. Reyes, Catherine A. Sarkisian

**Affiliations:** 1 USC Leonard Davis School of Gerontology, 3715 McClintock Avenue , Los Angeles, CA 90089 USA; 2 Department of Health Policy and Management, Fielding School of Public Health, University of California-Los Angeles, Los Angeles, CA USA; 3 Division of General Internal Medicine and Health Services Research, Department of Medicine, David Geffen School of Medicine at UCLA, Los Angeles, CA USA; 4 Section of Geriatric Medicine, Department of Medicine, Cedars-Sinai Medical Center, Los Angeles, CA USA; 5 David Geffen School of Medicine at UCLA, Los Angeles, CA USA; 6 RAND Corporation, Santa Monica, CA USA; 7 Geriatric Research Education Clinical Center (GRECC), VA Greater Los Angeles Healthcare System, Los Angeles, CA USA

**Keywords:** Latinos, Dementia, Antipsychotics, Alzheimer’s disease

## Abstract

**Background:**

Latinos are more likely than non-Latino Whites to develop dementia and be prescribed antipsychotics for dementia-related behavioral symptoms. Antipsychotics have significant risks yet are often overprescribed. Our understanding of how Latino caregivers of Latino older adults living with dementia perceive and address behavioral issues is limited, impeding our ability to address the root causes of antipsychotic overprescribing.

**Methods:**

We interviewed Latino older adults’ caregivers and community-based organization workers serving older adults with cognitive impairment (key informants), focusing on the management of behavioral symptoms and experiences with health services.

**Results:**

We interviewed 8 caregivers and 2 key informants. Caregivers were the spouses, children, or grandchildren of the older adult living with cognitive impairment; their ages ranged from 30 to 95. We identified three categories of how caregivers learned about, managed, and coped with behavioral symptoms: caregivers often faced shortcomings with dementia care in the medical system, receiving limited guidance and support; caregivers found community organizations and senior day centers to be lifelines, as they received relevant, timely advice and support, caregivers often devised their own creative strategies to manage behavioral symptoms.

**Conclusion:**

In-depth interviews suggest that the healthcare system is failing to provide support for behavioral symptoms from dementia; caregivers of Latino older adults rely on community organizations instead.

**Supplementary Information:**

The online version contains supplementary material available at 10.1186/s12877-024-05323-4.

## Introduction

The Latino population aged 65 years or older is projected to quintuple over the next four decades, rising to 22% of the older population in the U.S. by 2060 [1]. One of the critical issues facing Latino older adults is Alzheimer’s Disease and Related Dementias. Dementia describes a group of symptoms affecting cognitive function, memory, language, problem-solving, and social abilities [2]. Older adults with dementia can exhibit behavioral symptoms such as agitation, aggression, confusion, sleep disturbance, delirium, and walking about, which can be challenging for caregivers to experience and manage [3]. Many of these behavioral symptoms may be in response to unmet needs, such as hunger, thirst, pain, or lack of physical or social activity [4]. An estimated 1.3 million Latino older adults are expected to have dementia by 2050 [5]. Latino older adults are more likely to develop dementia than non-Latino White older adults [5]. Hypothesized risk factors driving this increased risk of dementia among Latinos include higher rates of type 2 diabetes, depression, hypertension, and cardiovascular disease [6] and lower levels of educational attainment [7–9]. Additionally, Latino older adults have earlier onset of dementia symptoms [10] and a higher prevalence of dementia-related behaviors such as hallucinations, combativeness, or wandering, compared to non-Latino White older adults [11].

Antipsychotics are psychotropic medications that are sometimes prescribed off-label to manage dementia-related behavioral symptoms such as agitation and aggression, however, these medications have limited positive effects and can increase cognitive decline, risk of falls, stroke, and death [12, 13]. The American Geriatrics Society strongly recommends that older adults with dementia avoid these medications unless non-pharmacological interventions have failed and the older adult is posing a threat to themselves or others [14]. Choosing Wisely, an initiative of the ABIM Foundation which promotes conversations between clinicians, patients, and caregivers about making choices to optimize high-quality care, notes that antipsychotics for dementia are regarded as low value care, i.e., care that is potentially unnecessary or harmful to patients [12, 15]. Low value care is described as medical services that should generally be avoided in favor of high value care, i.e., interventions, medications, or services which offer greater benefits compared to their risks. In the case of dementia, high value interventions and medications for addressing behavioral symptoms include non-pharmacological interventions such as reassuring disoriented individuals, reducing noise levels, providing stimulation with structured activities (e.g., music, crafts), addressing underlying pain or hunger, and providing physical activity throughout the day [16]. Second-line interventions include some classes of antidepressants, which have a safer risk profile for older adults.

Despite guidelines recommending against the use of antipsychotics in older adults with dementia, research shows they are frequently used in community dwelling older adults with dementia: more than one in five (21.6%) of older adults with dementia in the U.S. had at least one antipsychotic fill within the year [17]. Moreover, use of these medications is higher in Latino older adults compared to non-Latino Whites [18]. Latino older adults have a 1.62-fold greater use of antipsychotics compared to non-Latino White older adults [18]. Yet it is not understood why Latino older adults with dementia are more likely to receive antipsychotics than their non-Latino counterparts. Possible reasons for this disparity include low levels of dementia knowledge among Latino family members caring for older adults, low access to high-quality dementia care, or low levels of health literacy – defined as the ability to find, understand, and use information and services to inform health-related decisions [18, 19].

Informed and engaged caregivers can act as advocates for reducing low-value care – including antipsychotics – in persons with dementia. Yet we have limited understanding of how Latino caregivers perceive and understand various strategies to address behavioral issues in dementia. This gap in knowledge is a barrier to designing and implementing interventions aimed at addressing the overuse of low value care among Latinos older adults with dementia. The objective of this study was to explore the knowledge and experiences Latino caregivers of Latino older adults with dementia have around managing the behavioral symptoms of dementia as well as their experiences discussing medications for these symptoms with clinicians using a qualitative approach. Additionally, we sought to understand how Latino caregivers of Latino older adults with dementia managed their family member’s medications, and the types of conversations they had with medical providers around medication use. We also aimed to explore caregivers’ experiences with dementia-related health services to identify potential gaps in care. Understanding why Latino older adults are more likely to be prescribed antipsychotics for dementia-related behavioral symptoms can provide important background for designing interventions to reduce the use of these medications when possible.

## Subjects and methods

### Study design

We used a qualitative study design with semi-structured interviews conducted individually with Latino caregivers caring for Latino older adults as well as key informant interviews with caregiver advocates. Interviews were conducted in English and Spanish according to participant preference. Caregiver advocates included dementia support group leaders and senior day care center staff. We offered phone interviews to ensure that individuals with varying levels of digital literacy could participate. Additionally, as caregivers often have a difficult time finding respite care, phone interviews allowed caregivers to participate without leaving the home. Moreover, this study took place during the Covid-19 pandemic, which limited our ability to safely conduct interviews in person. We conducted interviews from October 2020-March 2021. We worked closely with a Community Advisory Board (the Los Angeles Community-Academic Partnership for Research in Aging, or LA CAPRA) as part of the UCLA Resource Centers for Minority Aging Research Center for Health Improvement of Minority Elderly (RCMAR/CHIME). We worked with the Community Advisory Board members to define the eligibility criteria, develop recruitment strategies, and design the interview guide. LA CAPRA includes leaders from community-based health organizations, community representatives, community development groups, and senior services organizations. The study was reviewed and approved by the Cedars-Sinai Medical Center Institutional Review Board.

### Study participants

Eligibility criteria for caregivers included: serving as a caregiver for a Latino family member either diagnosed with dementia or experiencing memory problems, being 18 years of age or older, and identifying as Latino. Per a recommendation from Community Advisory Board members, we interpreted the definition of the caregiver role broadly because it was felt that Latino families frequently share caregiving responsibilities among several family members.

We opted to interview key informants – caregiver advocates – as well as Latino caregivers in an effort to gain perspective of individuals who regularly interact with caregivers and learn about many challenges and experiences related to caregiving. Eligibility criteria for caregiver advocate key informants, who did not have to be Latino, included having regular contact with Latino caregivers of persons living with dementia in a professional context (e.g., serving as a caregiver support group leader or staff member at a senior day care center serving older adults with dementia).

### Recruitment

To recruit caregivers, we used several methods, including (1) flyers posted in geriatrics and primary care clinics at Cedars-Sinai Medical Center, a large health system in Los Angeles, CA with numerous outpatient clinics; (2) direct referrals from geriatricians and primary care physicians at Cedars-Sinai Medical Center; (3) flyers distributed through senior day care centers; (4) word of mouth (i.e., snowball sampling); and (5) emails and phone calls to caregivers participating in support groups associated with Los Angeles-area senior day care centers. We used a brief screening phone call or email to confirm eligibility and then scheduled the interview.

To recruit caregiver advocates, we relied on community partners in the Community Advisory Group to identify caregiver support group leaders and senior day care center staff in the Los Angeles area and emailed potentially eligible study participants. Study participants (caregivers and key informants) were offered a $60 gift card for their participation. We provided information sheets prior to the interviews via email and obtained oral informed consent from all study participants for this study.

### Interview guide

Our interview guide (Table 1, Appendix 1) for caregivers included a variety of topics structured around managing behavioral symptoms and experiences with healthcare services. We translated the interview guide into Spanish for the Spanish-language interviews using a professional translation service. MSK and NG, both fluent Spanish speakers, conducted the interviews in Spanish. For caregivers, we collected demographic information, including age, gender, race, ethnic background, educational level, and employment. We also collected demographic information about the person living with dementia and their caregivers. For caregiver advocates, we collected data on age, gender, race, ethnicity, and employment information. Interviews lasted 60–120 min and all were conducted by phone.

**Table 1:** Topics in the semi-structured interview guide discussed with Latino/a/x caregivers caring for a family member with dementia and key informant interviews with dementia support group leaders

Caregiver interview topics	Key informant dementia interview topics
• General experiences with caring for a family member with dementia • Experiences with medical care for the person with dementia • Managing medications for persons with dementia • Managing behavioral symptoms of dementia • Managing feeding/eating issues in persons with dementia • Quality of life • Preferences for educational interventions	• Perceptions of support and resources available to Latino caregivers • Recommendations given to family members around managing behavioral symptoms • Recommendations given to family members around managing weight loss/eating issues in persons with dementia • Discussions around end-of-life care with caregivers • Preferences about educational interventions

### Data analysis

All participants were asked for permission to audio-record the interviews. We audio-recorded the interviews and sent them out for professional transcription (and translation, if the interviews were in Spanish). Our study methodology was guided by Constructivist Grounded Theory (CGT) ([20, 21]. CGT is characterized by its inductive approach, where codes are constructed from the data and there is simultaneous data collection and analysis. After each interview, we refined the interview guide to explore potential lines of inquiry.

Two coders (NG and JAG) used line-by-line open coding to code all of the interviews in Dedoose, a qualitative coding software (Dedoose Version 9.0.17, (2021). Los Angeles, CA: SocioCultural Research Consultants, LLC) [22]. Line-by-line coding applies a code to every line of the data, allowing researchers to stay close (i.e., grounded) to the data [20]. Three investigators (MSK, NG, and JAG) met weekly for 10 weeks to discuss initial codes. All three members of the coding team were involved in discussions to create and refine the codes. Disagreements about the codes were resolved by reviewing the transcripts together in areas of disagreement and coming to a consensus both about how the code should be described and to which types of texts it should be applied. We used process coding [23], a method of coding which uses gerunds to describe actions in the data. For example, a line of text where a caregiver describes feeling frustrated with the jargon being used by medical professionals might be coded *Feeling frustrated with physician about complex language.* Process coding, used in conjunction with line-by-line coding, allows qualitative researchers to closely identify participants’ actions, beliefs, and experiences, reducing potential bias imposed by researchers [20].

After all transcripts were coded through this initial coding process, we exported all of the codes from Dedoose to an Excel spreadsheet and manually grouped the codes into focused, or second-level, codes, which are more conceptual in nature [20]. To maintain rigor, we discussed the creation of each focused code among the team. We then created the focused codes in Dedoose and categorized the initial codes under each focused code using the software. Finally, we grouped the focused codes into categories, discussing the nuances and complexities – referred to as *properties* in CGT – within each category.

In CGT, there is a focus on constant comparison throughout the analytic process [24, 25]. For example, we compared caregivers’ experiences with discussing behavioral symptoms with clinicians, developing strategies to manage behavioral symptoms, and sharing caregiving responsibilities with other family members. We met weekly throughout the analysis process to construct categories from the codes, noting down potential categories in written memos. Memo writing was used to record decision-making about initial code, focused code, and category construction; articulate potential nuances in each category; and communicate analytic decisions within the team [26]. Memo writing also allowed us to create an audit trail of our analytic process.

## Results

We interviewed eight caregivers and two key informants. Caregivers were the spouses (*n* = 2), children (*n* = 4), or grandchildren (*n* = 2) of the person living with dementia or experiencing memory problems, and caregivers’ ages ranged from 34 to 95 (Table 2). Our sample included two mother-daughter pairs caring for the spouse/father in the family where we interviewed both the daughter and the mother. Caregivers in our study identified as Peruvian, Salvadorian, Chicano, Mexican, and Venezuelan, and all resided in California (all but one resided in Southern California). Nearly all had some college education.

**Table 2:** Demographics of latino caregivers of person living with dementia who participated in the qualitative interviews

Caregiver alias	Caregiver gender	Caregiver age	Caregiver identity	Caregiver education	Caregiver relationship to PWD*	PWD gender	PWD age	PWD identity	PWD education
Adriana	Female	52	Peruvian	College graduate	Daughter	Female	98	Multiple races/ethnicities	< HS
Clara	Female	34	Salvadoran	Some college	Granddaughter	Female	88	Salvadoran	< HS
Roberto	Male	30	Chicano	College graduate	Son	Female	78	Mexican-American	< HS
Diego	Male	41	Salvadoran	Some college	Grandson	Female	88	Salvadoran	< HS
Soledad	Female	34	Mexican	Some college	Daughter	Male	76	Mexican	< HS
Maria	Female	57	Mexican	< HS	Wife	Male	76	Mexican	< HS
Paulina	Female	65	Venezuelan	Doctoral degree	Daughter	Male	95	Venezuelan	HS graduate
Olga	Female	95	Venezuelan	Master’s degree	Wife	Male	95	Venezuelan	HS graduate

*Notes: PWD: Person with dementia, HS: High school

Our key informants included a social worker (female, age: 50, non-Latina White) embedded in a community organization that provides adult day care services, and an activities coordinator at an adult day care center serving predominantly Latino older adults (female, age: 56, Mexican-American).

We identified three main categories of how caregivers learned about, managed, and coped with dementia and/or memory-related behavioral symptoms from our interviews: (1) caregivers often faced shortcomings with dementia care in the medical system, receiving limited guidance and support; (2) caregivers found community organizations and senior day centers to be lifelines in helping to manage symptoms, (3) caregivers often devised their own creative strategies to manage and minimize behavioral symptoms. Within each category, we identified sub-categories and describe them below. We use pseudonyms for the study participants in this manuscript to protect participant confidentiality.

### Facing shortcomings with dementia care in the medical system

#### Struggling with getting the diagnosis confirmed

Several caregivers described having a difficult time receiving a dementia diagnosis for their family member. Diego, 41, described that it took six months to get an initial evaluation with a neurologist to receive a diagnosis for his grandmother, making it difficult for him to get the appropriate services his grandmother needed. Clara, 34, also caring for her grandmother, reported laboring to find a doctor who believed her grandmother’s symptoms weren’t just old age. She described going to a primary care doctor to discuss her grandmother’s worsening symptoms, which included aggression and hiding things, and the doctor told her, *Oh*,* don’t worry*,* she just old.*

Two caregivers in our sample, 34-year-old Soledad and 57-year-old Maria, a mother and daughter pair, had several family members working in the medical system in both the U.S. and Mexico. In addition, Soledad and Maria regularly connected with family in Mexico to learn about the medications Soledad’s father/Maria’s husband was taking. This background empowered Soledad and her family to become more actively engaged in her father’s care. Despite this background, Soledad also struggled with getting specialty dementia care for their father. Soledad worried that her father had not yet seen a neurologist and explained that there were barriers that made it particularly difficult for her and her siblings to advocate for their father. First, she noted that her mother, Maria, who served as the primary caregiver for her father, did not speak English well so sometimes had a difficult time serving as an advocate. Second, she noted that she was located in Southern California while her parents were several hours away, and helped as much as she could, but she was not always able to accompany her father to the doctor’s appointments.

Interviewer: What is the process of diagnosis, what’s that has been like for [your father]? Soledad: It’s been a little rough because we definitely have been trying to get him better care. I know we’re trying to get him into a neurologist so that way they can do more activities, and I’m not sure if he’s seen like a therapist, psychologist… But I know it’s hard because we’re not 100% there to deal with the doctors directly…. But we’ve been trying to get him some extra help to see– so that way they can determine the actual condition. He’s with [health system] in [Central California] so it’s been a little tough trying to get things situated for him, because I don’t want it to get too far out because my dad’s dad had Alzheimer’s… We don’t want it to get to that severity where, you know, he doesn’t know where he’s at and he can get lost. We don’t want it to get to that level.

One of the key informants, Dina, a social worker at a senior day care center, confirmed the caregivers’ experiences, noting that many caregivers she met in support groups had family members who *haven’t specifically been diagnosed with Alzheimer’s disease. A lot of doctors are*,* you know*,* resistant to doing that.*

Dina: A lot of people come in and they’ve been seeing their primary care physician, and that could be someone they’ve been seen for 10, 15, 20, 30 years. And they trust them, but they’re not dementia savvy. And so a lot of them will say, Oh, this is just normal aging, or, Oh, they’re getting a little forgetful. And it takes them, the family members coming to the support group sometimes and sharing what’s going on, and the other members, as well as me telling them, You really need to ask for a referral to see a neurologist, to get some more, there’s some more going on than just, you know, normal age-related memory loss.

Julia, our second key informant, also described how clinicians working in smaller clinics or practices outside of large health systems weren’t as knowledgeable about dementia, making it difficult for family members to obtain a diagnosis. She described how the Latino caregivers she encountered often got little information about what to expect regarding the stages of dementia.

Julia: … the doctors in big hospitals, they know more or less how to find out that the patient is coming up with Alzheimer’s because they have more experience and, and they’ve been around more people. But, you know, the doctors in the small clinics, they don’t know much about Alzheimer’s.

She described how even when she advised Latino caregivers to advocate for their family members in these smaller practices, the clinicians were uncomfortable or upset about being questioned, particularly around the diagnosis of dementia or about prescribing medications such as cholinesterase inhibitors, thought to slow the progression of some dementia-related symptoms. She described how some doctors serving Latinos were not used to empowered patients or caregivers who asked a lot of questions:

Julia: And sometimes when the family knows… and I tell ‘em, You know what? She’s becoming very forgetful. Talk to the doctor and see if she can get some medication. Sometimes they even say that the doctors start laughing at that them. “Oh, so now you’re the doctor? So now you know what to prescribe to your mom or to your dad?”

Several caregivers said that they were concerned they did not have information about the progression of dementia and did not know what to expect. Clara described how she was worried her grandmother’s geriatrician may have been wary of preparing her for difficult days ahead, which she understood, but wished she understood what to expect:

Interviewer: Have you had conversations about either your grandma’s current behavior or what to expect with [the geriatrician] or anybody else?

Clara: No. [the geriatrician] just said that, you know, yes, she has dementia, but since my grandma really enjoyed, doing puzzles and being outside that she doesn’t think my grandmother will become that bad. She thinks that, you know, the forgetting things that’s, mixing up her days, the way she does, she said that that, that, that’s gonna be normal. But because my grandmother likes to do things a lot that perhaps we might not be there. But she might be sugarcoating it, I don’t know, not to freak us out, you know?

### Receiving medications for behavioral symptoms from clinicians, but little guidance on non-pharmacological therapies

Discussions with clinicians around behavioral and psychiatric symptoms were primarily centered on medication management. Several caregivers noted that their clinicians recommended medications for behavioral symptoms, including anti-depressants, benzodiazepines, and antipsychotics but offered few resources outside of medications. Maria explained that she had recently begun taking benzodiazepines prescribed to her by her doctor for anxiety and that she had given her husband a few pills to calm down his anxiety. When she found that they calmed him down, she talked to his doctor, who then gave him his own prescription, but soon had to add another medication to help with his anxiety:

Maria: I gave him one of my pills and I saw that he calmed down a bit and it helped him. And I made an appointment with his doctor and I told him and he said, Okay, let’s try them. He prescribed them to him and I have been giving them to him. But last year, even those weren’t working. So, that’s when I told all of these things to the doctor. I told her about his entire situation with memory and the stories that he would tell me over and over again… So, all of these things worried me and I told the doctor about them. So, I told her that the pills were not working anymore. So, she gave me some pills to help with his anxiety. So, I asked if I should take him off the other pills and she said, No, give him both. And then she prescribed some sleeping pills. So, that’s how we’re doing with that situation. But there are times when not even those pills work because he feels desperate, anxious, and restless.

Diego had a similar experience, where a physician prescribed quetiapine, an antipsychotic, for his grandmother’s sleeping issues and aggression but did not provide him with additional information on how to manage his grandmother’s behavioral symptoms. He found the process of managing his grandmother’s healthcare overwhelming and noted that it was difficult to absorb the information from his grandmother’s doctors. Likewise, Soledad described that she had received little guidance from her father’s physicians on how to manage her father’s anxiety and confusion and instead she and her family relied on their own approach for managing his symptoms.

Interviewer: Have any doctors or friends or family recommended any particular strategies that help when he gets like, with the questions, for example?

Soledad: Not really. No, hmmm, let’s see… Yeah, no. We just pretty much treat him as a kid, I mean, that’s we just have to pretty much think like you’re teaching a young child. In many occasions, we have to put our mindset that he’s at that level where we have to be extra careful and cautious of how we say things and just mild with a lot of things that we say to him.

Adriana, 52, who at the time of the interview was caring for her mother, described how her mother often got confused, for example, forgetting when she had already taken her medicine. Although she described that her mother’s physician was very attentive to caring for a variety of conditions, Adriana noted that she had not received any education on how to manage her mother’s confusion or other dementia-related behavioral symptoms.

Caregiver advocates confirmed these experiences. Dina, the social worker, described that in her experience, clinicians had little training or knowledge about how to help caregivers with behavioral symptoms and focused primarily on managing these symptoms with medications.

Dina: I think very, very few of them give alternative means of talk about managing behaviors. Usually they say send them to an adult daycare facility or place them. Um, a lot of doctors will say, well, if you can’t manage it, you know, maybe you should just pay for care or get a care, you know, get a caregiver in the home, that kind of thing. They don’t really go into educating the caregiver on how to manage behaviors. So, with the medication, they’ll talk to them about what the medication will do.

Julia, the activities coordinator at the adult day care center, noted that she spent a lot of time guiding family members about how to manage behavioral symptoms of dementia because she felt these were often unaddressed in the medical setting. She described coaching family members on how to manage wandering, hallucinations, and aggression, using strategies such as going with the flow, while avoiding arguing or telling the person living with dementia that they are going crazy. 

### Receiving little information about community-based resources from clinicians

Caregivers also mentioned that they often received few resources for caregiver support or training, for example information about feeding persons with advanced dementia. When asked whether the health system or physicians in the U.S. had given them resources on dementia care, Soledad explained:

Not really. We have doctors that have been suggested to us in Mexico, because my grandma currently has dementia. My mother’s mom has currently dementia and then my dad’s dad had Alzheimer’s, so we do have the resources to go to doctors in Mexico. My parents do travel two times a year, and they stay out there for a couple months. Other than that, no.

When resources were provided, Roberto, 30, who was caring for his mother, noted that it was a general sheet of resources and did not offer culturally or linguistically tailored community organizations or information. Roberto described how he had found an organization that provided culturally tailored caregiver support and had found it to be very helpful in providing him with education on how to manage the symptoms of dementia, but that he had had to find it on his own.

Interviewer: How did you get connected with the [organization] caregiver program? Roberto: I searched for a caregiver support group or I looked for a caregiving or maybe an adult day care center or similar, social service center that was Latino or something and I think that’s how I found the [organization] caregiver group….

Interviewer: And so some of the physicians that your mom sees, had they ever mentioned the existence of these kinds of groups before?

Roberto: No.

Paulina, 65, noted that in her experience, physicians in the dementia specialty center where her father was receiving care spent a lot more time measuring her father’s dementia progression and little time explaining how to actually manage the behavioral symptoms associated with dementia. She expressed her frustration with their focus on the assessments rather than more tailored information about resources that would be helpful for her father:

Paulina: The most that my dad’s doctor did was give them a list of resources. That’s the most that she did. The Alzheimer program is much the same way. I mean, the assessment was, oh my God this oh, incredibly long, Count backwards from 98 to one. I’m going to give you five words and in a few minutes I’m going to ask you to remember them. I mean, it’s pretty useless doing that every time and realizing that of course the person is getting worse. Of course the person is going downhill. A list of resources is … We had already found out about the daycare long before because I think the [city] prides itself in giving opportunities for everyone.

### Finding guidance through community organizations

#### Role of community-based resources in supporting Latino caregivers with dementia-related behaviors

Caregivers turned to other trusted sources of information for resources, including non-profit senior community centers and adult day care centers. For instance, Clara noted that she appreciated the emails and letters from the adult day care center her grandmother attended, which provided information and resources about dementia. The adult day care center staff also called her with updates about her grandmother and how to manage new symptoms.

Clara: [The senior day care center] does a really good job of sending out emails and little letters in case someone needs support. And thankfully, I haven’t had the need to or or felt like I really, really needed support. I’ve been doing pretty well with her. But I do feel like if there was that case, I could always talk to them, and the best part about it is that they’ve given me their cell phone numbers. So, you know, I can even call them and just say, Hey, you know, she did this, you know?

Paulina and Diego both noted that the senior community center their family member attended became a lifeline. Paulina described that the community center provided both culturally competent subsidized care for her father and a resource for her and her mother in terms of helping them cope with dementia.

Paulina: It became very clear to us that [the senior community organization] was the most important thing in his life…. [the Latino caregiver services staff member] would tell me too, Oh, your dad was especially confused today. Just wanted to let you know. Your dad did this today. He didn’t want to play dominoes with us. He didn’t even want me to help him. They knew of his gradual decline as well, because they would see him. The [center director] is always available to me. [The center director] always knew that she could call me. And then [the Latino caregiver services staff member] gave me her cell phone number.

Dina, the social worker, described how some physicians who were knowledgeable about community-based resources were able to refer family members struggling with behavioral issues to support groups, where they often received guidance both from support group staff and from people who were caring for persons living with advanced dementia.

Dina: And so a lot of reasons why they come into caregiver support group, is– I would say the majority of people came because either their physician – good physicians, right -- or their family members, like their adult children or friends or siblings, or, you know, family members have strongly suggested they go to care group a support group because they are seeing that they’re not handling the behaviors very well. And so they’re seeing that they’re getting frustrated and not accepting the reality of where their loved one is at, and really trying to kind of, um, push them.

### Using creative strategies to manage and minimize behavioral symptoms, but struggling with how to manage trauma-related symptoms

Caregivers in our sample described that in the absence of much guidance from the healthcare system, they experimented with many different types of activities in order to reduce anxiety, depression, and agitation in their family member. Caregivers also reported how difficult experiences and trauma in their family members’ lives made them anxious or depressed, which was challenging for caregivers.

#### Creating activities and strategies independently to manage behavioral symptoms of dementia

Caregivers reported myriad behavioral symptoms in their family member with dementia, including disorientation, aggression, hallucinations, agitation, problems with sleep, confusion, and memory loss. Caregivers noted that they independently devised and tried a variety of creative ideas to reduce agitation and improve mental engagement, such as playing their family member’s favorite music, serving their family member their favorite foods from their home country, initiating games of dominoes or bingo, offering coloring books and puzzles, offering children’s books, and connecting their family member to other relatives on the phone.

Maria: Oh, yes, I play music for him. I do that. And he does become more relaxed with music. One of his daughters bought a record player to play the records he likes there. He plays records there. Or I play music for him on my phone. He likes listening to the radio.

Nearly all caregivers noted that they used strategies often used with children, such as using simple language when explaining things and using time outs when the person with dementia was feeling agitated or upset.

Clara: She is an adult, but I also feel that her brain is becoming back like a child. So, sometimes you need to put them on a timeout. So I tell her, You know what? I see that you’re very sad. I think… Or I see that you’re very upset, you know? Whatever the emotional issue is, I’ll tell her, Take some time out, go to your room and just relax, just relax. And when you’re ready to come back out, you know, then we can talk about it, you know, because it’s not good to, to talk about things when you’re emotional. You know, and then she’ll go to the room, and she’ll sit there, and I’ll tell her to do some coloring to get her mind off of it.

#### Finding it challenging to help family members with dementia address experiences of trauma earlier in life

Multiple caregivers spoke about their family member’s experiences with trauma earlier in life, often due to poverty or political strife, and how it affected their family member’s mental health. Caregivers found it challenging to help their family members manage symptoms related to anxiety and depression related to their family members’ traumatic experiences. Clara described how her grandmother in El Salvador witnessed traumatic events, such as seeing her husband and son killed in front of her eyes. She found it challenging to know how to respond when she found her grandmother crying at night due to these painful memories.

Clara: I have noticed that at times, at night, … sometimes I’ll see her cry at night. And, and I’ll say, Hey, what’s wrong, you know? And then she’ll say, Oh, I’m just thinking about how old my son would be or, or where we would be right now with my husband? And, you know, she never remarried. Um, and so I think that, that affects her.

Maria also noted that her husband spent a lot of time talking about his difficult childhood, making it challenging for her to soothe his anxiety and distress. She didn’t know how to respond to his sadness and anxiety around this trauma, which caused her much distress.

Maria: And since he had a lot of problems with his father during his childhood, he’s carrying all of those issues. He’s repeated that to me many times and he still repeats it a lot. There were beatings and also financial struggles. So, they went hungry. So, he tells me all of those things. And he tells me a lot about an uncle that helped him a lot and he passed a while back. I tell him, ‘That’s good. That means that your father was really this man, your uncle. It’s like he’s not really gone because he’s on your mind. That’s good, that you remember.’ So, he talks and talks and all of that.

## Discussion

In this exploratory qualitative study of eight Latino caregivers and two key informants who work closely with caregivers, we identified three main categories describing approaches to managing behavior related to dementia symptoms and interacting with the healthcare system: caregivers (1) often face shortcomings with dementia care in the medical system, encountering delays to diagnosis, barriers in accessing neurologists and other specialists, and clinicians who dismiss symptoms of dementia as old age or who bristle at being questioned; (2) find resources and support from community based organizations, but receive little information from healthcare providers about these organizations; and (3) independently formulate creative strategies to manage behavioral symptoms in the absence of guidance, but find it challenging to manage symptoms associated with trauma. Although caregivers in our sample had different backgrounds and experiences, we found several commonalities with regards to their experiences caring for a family member with dementia. We found that regardless of knowledge or prior exposure to dementia, all caregivers in our sample wanted better access to resources to help them as caregivers. Our findings are consistent with literature that Latino caregivers are eager to learn more about dementia care and are aware of the importance of increasing their level of knowledge about the conditions [27–30].

We found that caregivers overall felt unprepared for managing their family member’s dementia diagnosis, even among caregivers with high levels of health literacy. This echoes other studies findings that many families feel ill-equipped to manage such a complex progressive diagnosis [28, 30]. Other research finds that even when family members are aware of symptoms and have some familiarity with dementia, they do not always feel adequately equipped with how to address challenging situations, such as changes in the patient’s sleep or increasing levels of anxiety [31]. Moreover, many behavioral symptoms of dementia may be related to an inability to express unmet needs, such as hunger, thirst, loneliness/social isolation, boredom, and lack of physical activity [4]. Pain is also commonly the underlying cause of behavioral symptoms associated with dementia, particularly among persons with more severe cognitive impairment [32, 33]. Caregivers may not be aware of how to identify these unmet needs or how to respond to challenges surrounding eating and drinking in the later stages of dementia [34].

Complicating caregivers’ experiences with dementia care is the fact that many primary care clinicians are not comfortable or trained in how to diagnose or manage individuals with dementia [35, 36]. Several caregivers in our sample had a difficult time obtaining a diagnosis and found that clinicians dismissed their family members’ symptoms as old age, findings which other qualitative studies of caregiving (not limited to Latinos) have confirmed as well [27, 28]. Our findings echo a 2019 Alzheimer’s Disease International report which surveyed 70,000 respondents across 155 countries that found that 62% of healthcare practitioners worldwide still perceive that dementia is a normal part of aging, which results in significant barriers to the appropriate diagnosis, treatment and care for persons living with dementia and their caregivers [37]. Moreover, prior research finds that primary care providers report limited time to address the complex management of dementia or have little knowledge about the dementia diagnostic process [38, 39], and that caregivers report receiving inadequate support from their providers in managing dementia-related problems [34, 40]. Moreover, primary care providers report having little connection to social services, community organizations, or interdisciplinary teams which could assist caregivers with dementia management [38, 41]. These factors can make for unsatisfactory encounters between persons with dementia, caregivers, and clinicians, and can result in caregivers feeling underprepared and dissatisfied with how the medical system manages dementia. Future research should examine how to design better care experiences for persons with dementia and their families within the current medical system.

Our findings shed light on some of the challenges faced by Latino caregivers as they look to clinicians for assistance on managing behavioral symptoms of dementia. When asked about conversations with clinicians to manage dementia-related behavioral symptoms, caregivers in our sample often brought up discussions with clinicians about the use of medications to treat sleeping problems, anxiety, depression, and agitation. One reason for prioritizing medications over non-pharmacological therapies may be the medical system’s approach to health as informed by a cure model, where a biomedical approach is prioritized over a psychosocial approach (i.e., the care model) [42–44]. Even as some primary care providers are aware of the limits of the biomedical approach when it comes to dementia care, research has found they feel frustrated with the fact that there is little that medicine can do to alter the progression of the disease [39]. As a result, one study found that primary care physicians still regularly prescribed medications for dementia – even when they found them to be lacking in effectiveness – because they wanted to help patients and their families in some way [39]. Clinicians may perceive that medications are some of the few tools they are able to offer families struggling with managing difficult behavior symptoms. Additionally, health insurance covers medications, but does not routinely cover caregiver support. Training clinicians in how to offer tangible strategies for managing behavioral symptoms may provide primary care providers with additional tools they can offer families. However, offering such support can be challenging during a 10 to 15-minute primary care visit. Connecting caregivers to community organizations may be able to fill in gaps which primary care providers are not currently able to support.

Indeed, we found that caregivers in our sample found a great deal of support through community-based organizations and also their own families and solutions. Several interventions have been developed specifically tailored for Latino older adults living with dementia and their caregivers, including radio-based support groups [45], bilingual educational websites [46], telephone-based support groups [47], *promotora-*based community engagement [48], and culturally and linguistically tailored programs [49–51]. However, as we found in our study, linking caregivers to these types of programs and resources remains a challenge, as many health system-based clinicians and social workers may not have knowledge or updated lists of culturally and linguistically appropriate services. One promising intervention is the Care Ecosystem Model, a model which connects family caregivers with care navigators [52, 53]. This model has been found to improve the quality of life in persons with dementia and decrease caregiver depression and burden [52]. Tailoring and disseminating the model to communities which serve a large proportion of Latinos has the potential to provide to substantial support to many caregivers.

Despite little guidance from medical professionals, caregivers in our sample found creative ways to engage their family members in meaningful and pleasurable activities such as playing music, offering coloring books and puzzles, or caring for pets. These meaningful activities may play an important role that goes beyond enjoyment: Nyman and Szymczynska found that these activities can meet fundamental psychological needs [54]. For example, these activities may give individuals a sense of control, address the need to be creative, and strengthen relationships and social connectedness [54, 55]. Engaging persons with dementia in meaningful activities can also be critical for caregivers. An ethnographic study using interviews and participant observation found that when caregivers discontinue activities meaningful to persons living with dementia, caregiver burden increases substantially [56]. However, caregivers can adapt or replace activities as the dementia progresses, if provided the right training and social support. Supporting caregivers in adapting activities to changing barriers, such as the development of vision and hearing loss, as reported by several caregivers in our sample, is critical to both the progression of cognitive decline and caregiver health. Epidemiological studies have found that untreated vision and hearing loss can increase the risk of cognitive decline and dementia and accelerate the progression among those already living with dementia [57–59]. This research points to an even greater need to support caregivers who are caring for older adults with vision and hearing loss as well as dementia and associated cognitive decline.

One striking finding was the high prevalence of trauma in early life among the Latinos living with dementia in our sample. Some studies have found an association between experiencing several adverse childhood experiences such as parental death, family violence, physical or psychological abuse and dementia [60], and others have found a bidirectional relationship between post-traumatic stress disorder (PTSD) and dementia, where PTSD may increase the risk of dementia and the onset of dementia can increase the risk of delayed PTSD [61]. Persons living with dementia may experience traumatic flashbacks [62], which can be alarming for the person living with dementia and their caregiver. Symptoms of PTSD may be confused for behavioral symptoms of dementia [63]. However, caregivers and clinical staff made aware of the person’s past and potential triggers for the PTSD can employ strategies to reduce stress and agitation among persons living with dementia who have experienced traumatic events. A study of nursing assistants’ experiences caring for older people with dementia who experienced Holocaust trauma found that understanding the person’s life story enabled adjustments in care and gave the nursing assistants greater empathy and patience in caring for the person with dementia [64]. Training caregivers in how to manage PTSD in family members with dementia who have experienced traumatic events may reduce the use of psychoactive medications and improve caregiver burden.

There are several limitations in our study. First, Latinos as a group are extremely heterogenous and Latino ethnicity encompasses individuals from numerous countries and backgrounds. Additionally, many of the issues we identified are likely not unique to Latinos. Moreover, U.S. Latinos’ experiences may be largely shaped by the areas where they are living. Our findings are thus more generalizable to Latinos living in the Southern California area, where the majority of our sample was from. We also had a small sample size. However, despite this small sample, we found similar experiences among caregivers in our sample, and these experiences have been echoed in other qualitative studies of Latino caregivers of Latino older adults living with dementia. Moreover, several studies have found that 10 interviews typically produce the majority of salient themes in interview-based analyses [65–67]. Future research should examine similar questions with a larger sample or with Latinos in different regions of the country.

In conclusion, in this exploratory study of Latino caregivers’ experiences managing behavioral symptoms of dementia, we found that caregivers struggled with their family members’ dementia-related behavioral symptoms, including aggression, anxiety, depression, and sleeping difficulties. Caregivers also reported that clinicians often offered medications for behavioral symptoms. Caregivers found a great deal of support in community-based organizations, including senior day care centers, caregiver support groups, and intergenerational community centers. In-depth interviews with this small sample suggest that the healthcare system is failing to provide care for behavioral symptoms from dementia so that caregivers of Latino older adults rely on community organizations instead.

### Electronic supplementary material

Below is the link to the electronic supplementary material.

Supplementary Material 1

## Data Availability

The datasets generated and/or analysed during the current study are not publicly available due to their identifiable nature but are available from the corresponding author on reasonable request.
