# Supplementary material for: Experiences managing behavioral symptoms among Latino caregivers of Latino older adults with dementia and memory problems: a qualitative study
Source: BMC Geriatr. 2024 Aug 31;24:725. doi: 10.1186/s12877-024-05323-4 (PMC11365257; doi:10.1186/s12877-024-05323-4)
Supplement: Supplementary file 1 — Supplementary Material 1 [file 12877_2024_5323_MOESM1_ESM.docx]

**Experiences managing behavioral symptoms among Latino caregivers of older adults with dementia and memory problems: a qualitative study**

**Appendix 1. Semi-Structured Interview Guide**

**Experiences of caregiving & interactions with medical system**

1. Can you tell us a little bit about yourself and the person who is experiencing memory problems or problems thinking?
2. What are some of the ways in which you help to take care of your [wife, husband, mom, dad, friend]?
3. Are there any other medical issues your family member is experiencing?
   1. *Prompt: such as diabetes, heart disease, or heart failure*
4. Where does your [wife, husband, mom, dad, friend] typically get medical care?
   1. Is this the doctor(s) that manages your family member’s memory care? Can you tell us about the doctors or other providers you have met with and talked to about your family member?
      1. What have those conversations been like?
      2. What was the last visit like? What did you talk about?
5. Do you manage the medications for your family member?
   1. What has that been like?
   2. What sorts of medications do they take?
   3. When you have a question about a medication for your family member, who do you go to?
   4. Has your family member had any problems with the prescribed medications?
   5. Does your family member take any alternative or unprescribed medications such as herbs or supplements?
6. Can you remember a time in the past when your doctor stopped one of the medications for your [mom, dad, friend]?
   1. Do remember why it was stopped?
   2. If the medications were adjusted, what happened?
7. Are there any medicines that you think your [mom, dad, friend] with memory problems might not need anymore?

**Managing behavioral symptoms of dementia**

1. Now I want to talk a little bit about symptoms people with memory problems might experience. Sometimes people can experience confusion and agitation - is this something that your loved one has experienced?
   1. Can you tell me about the last time this happened? What did you do?
      1. Did that work? If not, what did you do next?
      2. Did you use urgent or emergency care?
   2. What have others recommended that you do?
      1. *Prompt: Have you had conversations with friends or family about what to do? What did they recommend?*
      2. *Prompt: Has a doctor or nurse recommended anything that you could do?*
      3. *Prompt: Have your doctors or others recommended any sort of medications or other strategies that can be used to help manage this type of agitation?*
         1. What do you think about these kinds of medications?
   3. Has this agitation/confusion ever happened in the hospital or any sort of medical facility?
      - 1. If yes, can you describe what happened?
        2. Did the doctors or nurses recommend any sort of physical restraints? If so, can you tell us about this experience? What happened?
2. Has your family member struggled with losing weight or not eating?
   - 1. If yes, what have you tried, if anything? What happened next?
     2. If yes, has a doctor or nurse recommended any strategies to make feeding your family member easier?

**Palliative care**

1. Tell me about your loved one’s quality of life. Does your loved one experience any issues that impact their quality of life?
   1. *To what degree is your loved one healthy, comfortable, and able to participate in or enjoy life?*
   2. *Prompt: Does your loved one experience any symptoms or issues that you believe might need better treatment?*
2. Are you able to talk to the doctor about your loved one’s quality of life? What are these conversations like?
3. Have you ever met with a doctor who specializes in treating the symptoms and side effects of your loved one’s memory problems? They might have been called a “palliative care” or “supportive care” doctor.
   - 1. What was the last visit like? What did you talk about?
4. Do you feel that you are getting the support you need as a caregiver?
   1. *Prompt: What do you feel is missing or lacking?*
5. If you could have anything you wanted to help improve your loved one’s quality of life, what would it be?

**Preferences for interventions**

1. When it comes to caring for someone with memory problems, what would be helpful to learn more about?
2. Where do you typically find information about caring for someone with memory problems? What have been helpful sources?
   1. *Prompt: (friends, family, doctors, church, support groups, social media)?*

**Experiences with racism in the healthcare system**

1. Do you think doctors of your own race/ethnicity explain treatments differently than others who are not? In what way?
2. Have you ever experienced feeling/thinking like you were not offered optimal care because of racism or prejudice?
   1. What was that like, if you feel comfortable sharing?

**Conclusion**

1. Is there anything else you would like to mention that we didn’t ask you about?
